# Monocular Vision-Based Coastal Distance Estimation for GNSS-Denied AUV Operations: A Feasibility Study

**DOI:** 10.3390/s26216713

**Published:** 2026-10-23

**Authors:** Jacek Zalewski

**Affiliations:** Department of Computer Science, Polish Naval Academy, ul. Śmidowicza 69, 81-625 Gdynia, Poland; j.zalewski@amw.gdynia.pl

**Keywords:** autonomous underwater vehicle, monocular vision, distance estimation, image segmentation, coastal environment, GNSS-denied navigation, reference object

## Abstract

The issue discussed in this article concerns navigation support, including that for autonomous underwater vehicles, which are subject to a systematic increase in positioning error when using dead-reckoning methods in GNSS-denied coastal areas. In such situations, the ability to capture images of the vehicle’s surface surroundings and use information about objects within the field of view of the optical system becomes useful. The proposed method uses a monocular camera and image segmentation to estimate distances to selected coastal reference objects. Determining the number of pixels occupied in the vertical plane by an object of known height enables the distance to be calculated using well-known mathematical formulas. The method was evaluated as a semi-automatic feasibility study using images acquired from a motorboat under maritime conditions. Forest regions of interest and the sea–land reference line used for building measurements were selected manually. The resulting estimates were evaluated against adopted reference distances derived from camera GPS coordinates and manually selected mapped reference locations. The reported differences describe agreement with these references, whose uncertainty was not independently quantified, and do not establish metrologically traceable absolute measurement accuracy. Image quality and segmentation errors affect the resulting distance estimates. The results indicate the potential of the method to provide auxiliary distance observations; however, deployment on an AUV and integration with its navigation system were not experimentally validated. This article describes the method and presents the results.

## 1. Introduction

Autonomous Underwater Vehicles (AUVs) are becoming excellent tools for marine exploration. Their widespread use allows them to perform tasks that, until recently, required human personnel. They are usually equipped with various sensors, such as sonar or cameras, that allow them to monitor their surroundings. They can find their way around complex underwater environments, using special algorithms and navigation systems.

AUVs face many navigational challenges due to the underwater environment [1]. The lack of GNSS signals significantly impacts navigation accuracy. It is also complicated by ocean currents and a flattened bottom. The methods currently used for AUV navigation include dead reckoning, inertial navigation, acoustic navigation, and geophysical navigation [2]. The navigation errors accumulate over time, especially when relying on low-cost IMUs [3]. In dynamic and uncertain underwater scenarios, local path planning and obstacle avoidance are important for ensuring the safety of an AUV. Recent research focuses on a multi-source sensor fusion framework for AUV localisation. The vehicle position can be obtained from Beacon Passive Acoustic Navigation systems [4]. When radio-navigation signals are unavailable or jammed, dead-reckoning error increases with time [5]. AUVs need a method that estimates position from onboard sensors.

Unmanned vehicles are typically equipped with onboard cameras. While their underwater range is limited, cameras are valuable for surface observations. Image processing algorithms enable the extraction of useful information from images. Segmentation is the process of partitioning a digital image into several regions with similar properties. Both traditional algorithms and deep learning convolutional neural networks are used for this purpose. In autonomous navigation, image segmentation enables interpretation of the full scene context by providing pixel-level object classification.

The proposed method is intended to use monocular images acquired during temporary AUV surfacing. Image segmentation is used to identify coastal objects, such as forests and tall buildings. Based on the pinhole camera model, the distance to each reference object is estimated from its known height and its corresponding pixel height in the image. The method provides passive distance estimates from individual monocular images and does not independently determine the vehicle’s position. When associated with a geographically identified reference object, such a measurement could constrain position uncertainty in the radial direction relative to that object. Full three-dimensional reconstruction, visual SLAM, active target tracking, and integration with inertial navigation are outside the scope of this feasibility study.

The proposed distance-estimation method was evaluated under real-world maritime conditions. Its estimates were compared with adopted reference distances obtained in Google Earth from camera GPS coordinates and manually selected coastal reference locations. This comparison evaluates agreement with the adopted references rather than independently validated absolute measurement accuracy.

The main contribution of this paper is:

- A monocular vision-based method is introduced for estimating the distance from coastal objects;
- Two categories of reference objects are investigated: forest and building;
- The proposed method is tested under real-world maritime conditions;
- Limitations of the method are identified.

## 2. Related Work

This section reviews recent advances in navigation techniques for positioning and obstacle avoidance for autonomous vehicles. The first of these is terrain-based navigation (TBN), in which the recorded vehicle’s surroundings are compared with the map [6]. Vision-based navigation (VBN) uses visual information from camera images for localisation and motion estimation. Depending on the approach, it may use a pre-existing map, construct a map during operation, or operate without an explicit map. Its performance can be affected by low visibility, variable illumination, and a lack of distinctive visual features [7]. Recent research in this area has focused on deep learning methods [8], including semantic segmentation, object detection, vessel tracking, re-identification, and multimodal sensor fusion.

The existing reviews of terrain-based and terrain-aided underwater navigation summarise sonar-based approaches and their principal challenges [9,10]. Broader reviews address obstacle avoidance in UAVs [11], self-contained localisation [12], AUV localisation and navigation [13], and vision-based navigation [14]. Maritime perception is considered through situational awareness [15], sonar-image analysis [16], and marine object detection [17]. Further surveys cover visual odometry [18], learning-based perception and navigation [19], image-based obstacle detection [20], and AUV navigation strategies [21]. These studies provide the broader navigation context. For the present work, the more specific question is whether the apparent vertical extent of coastal reference objects in individual monocular images can provide useful distance observations under maritime conditions.

Paper [22] presents a monocular-camera distance estimator, called Vessel Object-Focused Distance Estimation, which uses instance segmentation to extract vessel dimensions and improve distance-estimation accuracy. The method is suitable for low-cost maritime platforms and surveillance applications in the absence of GNSS signals. Article [23] presents distance estimation to other vessels using a single camera. The advantages of this approach include the use of an inexpensive camera and the fact that the size of the observed vessel need not be known. For ground vehicles, distance estimation for monocular images that integrates measurements to calculate vehicle position, velocity, and time to collision is shown in [24].

The article [25] explains how autonomous surface vehicles use camera images and semantic segmentation to detect and avoid obstacles. First, a convolutional neural network (CNN) extracts features and detects the horizon; then classical vision algorithms identify obstacles and plot safe routes. The paper [26] proposes an image-segmentation-based obstacle-detection system for ASVs. Another method, presented in [27], uses a deep neural network to segment water regions for USVs operating in unknown environments. The method in [28] divides images into two types and applies an adaptive threshold or a two-category region classifier. Previous studies [29,30] investigated coastal-position estimation by comparing image-derived representations of the surroundings with map-derived representations at candidate-vehicle positions. The present study evaluates a different measurement task: estimating the distance to a selected coastal reference object from its apparent pixel height and an assumed or independently obtained physical height. The output is an auxiliary range observation rather than a vehicle position obtained through map matching.

The present study focuses on the experimental applicability of height-based monocular ranging to coastal reference objects. Two complementary cases are examined: forests, whose physical height must be approximated, and buildings, for which independent height information can be obtained but the base may be occluded. The contribution lies in evaluating these cases using maritime images across different observation distances and examining how reference-height assumptions, segmentation-derived pixel measurements, and visibility limitations affect the resulting estimates. The underlying pinhole projection model is established; the study assesses its practical capabilities and limitations within a semi-automatic coastal-ranging procedure.

## 3. Methodology

The method described in this article investigates the potential of monocular imagery and image segmentation for estimating distances to selected coastal reference objects. The distance to an object of known height can be determined by measuring the number of pixels it occupies in the vertical plane and then applying well-known formulas.

In the envisaged AUV application, images would be acquired during temporary surfacing. In the present experiments, images were acquired from a motorboat using a handheld camera positioned approximately 1 m above the water surface. Computer-vision techniques and image segmentation were used to identify areas occupied by specific types of land-based objects. Two categories of reference objects were considered: coastal forests, for which an approximate average height was assumed, and tall buildings, for which independently available height information was used.

The proposed processing procedure comprised the following stages:

- Acquire a coastal image;
- Locate the reference object;
- Select the region of interest corresponding to the reference object;
- Determine the apparent vertical object height in pixels;
- Set the real-world reference height;
- Estimate the distance to the object;
- Compare results with GNSS-based reference distance.

The experiments presented in this paper focus on distance estimation. Automatic landmark association, multi-object triangulation, vehicle localisation, and integration with other navigation are not addressed in the present study; these topics will be examined in future work.

### 3.1. Image Acquisition

**Figure 1:**
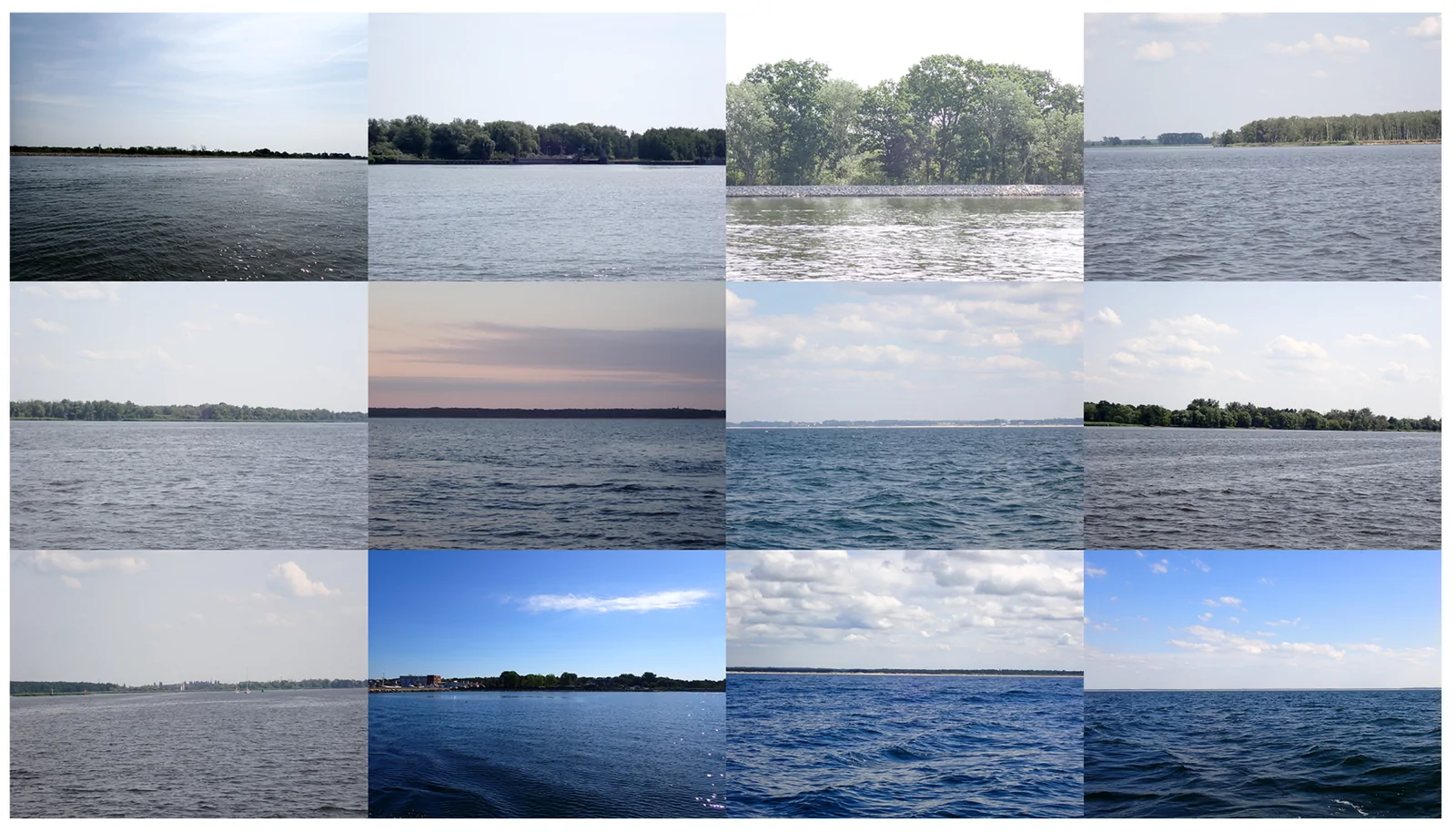
Examples of coastal images acquired under different observation distances, illumination conditions, visibility conditions, and coastline configurations.

Accurate distance estimation depends on high-quality image acquisition. Selecting appropriate imaging equipment and settings is essential for reliable feature extraction and analysis. Resolution, focal length, and preprocessing techniques influence measurement accuracy. The following subsection details the camera specifications and settings used in this study. Shoreline images were captured at distances from approximately 200 to 12,000 m offshore (Figure 1).

All experimental images were acquired using an Olympus TG-6 camera. The images used in the experiments had a resolution of $N_{x}\times N_{y}=4000\times 3000$ pixels, where $N_{x}$ and $N_{y}$ denote the horizontal and vertical image dimensions, respectively. The physical sensor dimensions were $w_{s}=6.17\;\mathrm{mm}$ and $h_{s}=4.55\;\mathrm{mm}$. All images analysed in this study were acquired at a fixed nominal focal length of f=6 mm, as confirmed by the EXIF metadata. The images were acquired from a small motorboat using a handheld camera positioned approximately 1 m above the water surface. An effort was made to maintain a consistent observation height. Camera attitude was not recorded, and no IMU-based attitude compensation or gimbal stabilisation was applied. Consequently, variations in camera orientation and height caused by boat motion were not quantified.

The camera positions were obtained from the GPS coordinates stored in the image EXIF metadata and imported into Google Earth. Reference distances were measured in Google Earth between each camera position and the corresponding coastal reference location. The coastal reference locations were selected manually. For forest measurements, the reference point corresponded to the shoreline location at the centre of the selected ROI. For building measurements, the mapped location of the reference building was used. The accuracy of the camera GPS positions and the manually selected coastal reference locations was not independently quantified. The resulting reference distances, therefore, have unquantified uncertainty. The integrated electronic compass identified the approximate viewing direction and corresponding coastal region. No explicit geometric camera calibration was performed before the experiments. The distance calculations used the camera parameters specified above. In Phase 2, the analysed regions were located close to the optical axis to reduce the potential influence of lens distortion. This arrangement did not replace camera calibration, and the remaining distortion was not quantified independently. Consequently, the observed differences between estimated and reference distances reflect the combined effects of geometric-model limitations, reference-height uncertainty, image-measurement errors, and uncertainty in the adopted reference distances. These contributions were not quantified separately, and the comparison does not establish metrologically traceable absolute measurement accuracy.

### 3.2. Image Segmentation

The YOLOv8x-seg instance-segmentation network  [31] was used to generate object masks. Masks of detected instances belonging to the same class were combined into a class-wise binary mask for subsequent image measurements. The network was initialised using publicly available pre-trained weights and fine-tuned on a dedicated dataset comprising approximately 10,000 high-resolution images from coastal and maritime environments, captured at various observation distances and under different illumination, visibility, weather, and sea-state conditions. Additional publicly available images were obtained from Roboflow Universe [32] to increase dataset diversity. All segmentation masks were manually annotated. The manually annotated dataset was divided at the original-image level into training and validation subsets containing 80% and 20% of the images, respectively, before tiling. Tiles were generated during training-data processing, with all tiles derived from a given image retained in the same subset. Separate images, excluded from both subsets, were used in the distance-estimation experiments. Forest and building masks provided the reference-object measurements, while sky and sea masks supported scene separation.

During training-data processing, images were resized and divided into overlapping tiles. The network input size was 640×640pixels. During inference, images were also processed as overlapping tiles using the same network input size, and the resulting masks were reassembled in the original image coordinate system. Overlapping helped preserve small and distant coastal objects and reduced the likelihood of splitting objects at tile boundaries.

The YOLOv8x-seg network was fine-tuned for fifty epochs. The principal training parameters are summarised in Table 1.

Automatic optimiser selection was enabled in the training framework using the setting optimizer=auto. Data augmentation was used to improve the network’s generalisation capability, including changes in hue, saturation, and brightness, as well as random translation, scaling, horizontal flipping, mosaic augmentation, random erasing and an automatic augmentation policy. The corresponding parameters are presented in Table 2.

Table 3 reports the class-wise mask average precision at an IoU threshold of 0.5 on the validation subset for the four classes used in the distance-estimation procedure.

**Figure 2:**
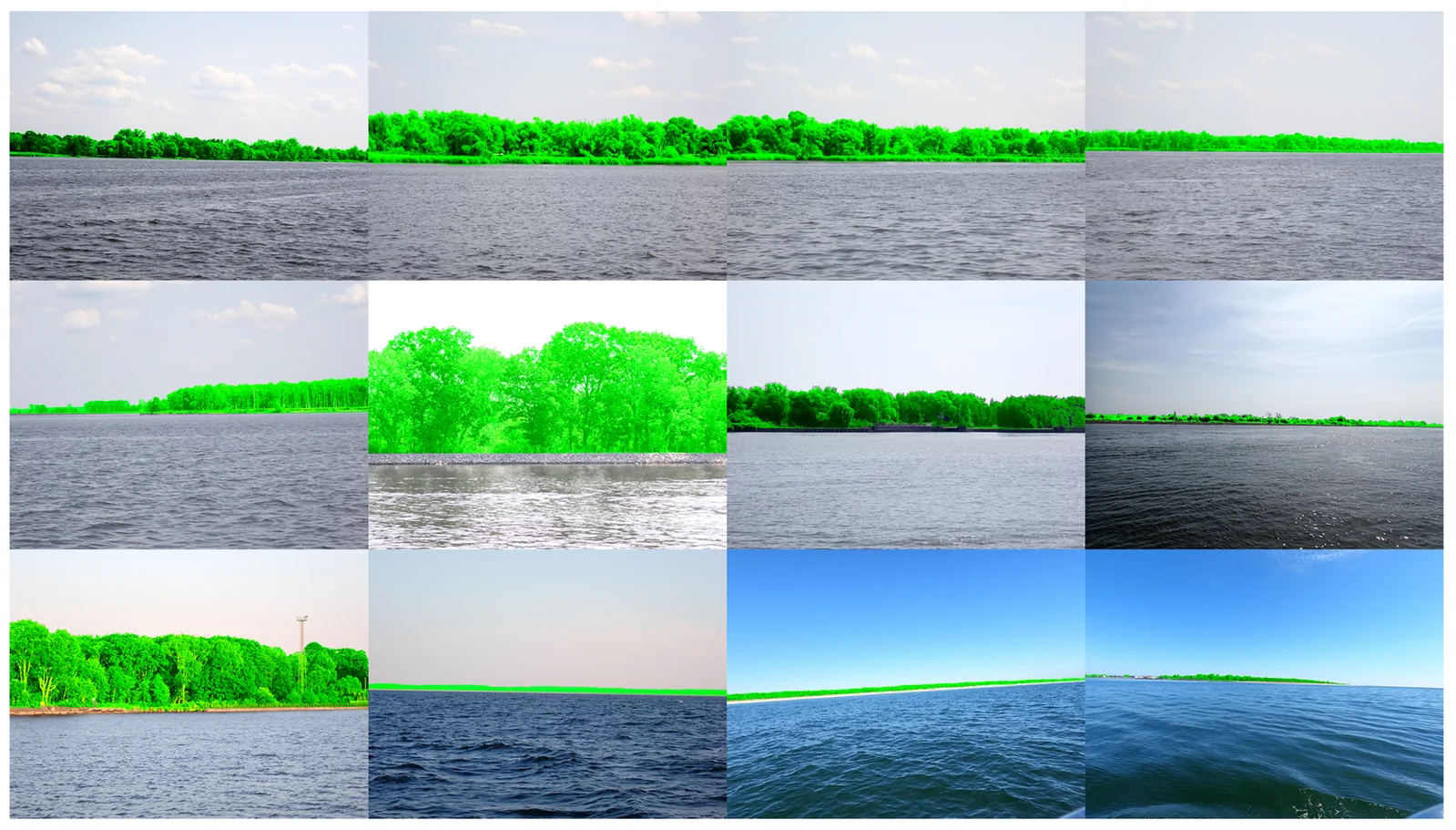
Examples of image segmentation results for coastal images. The forest class used for the subsequent distance-estimation procedure is highlighted in green.

The reported AP values summarise performance on the validation subset. Separate quantitative evaluations by observation distance, illumination, haze, and sea state were not performed (Figure 2).

Camera Specifications and Settings

For forest-height-based experiments, the segmentation mask for coastal vegetation was used to determine the vertical extent of the reference object. A 56-pixel-wide region of interest was selected manually to define the coastal sector analysed in each image. This allowed the segmentation and geometric ranging stages to be evaluated without introducing errors from automatic ROI selection and landmark association. For building measurements, the sea–land reference line was also selected manually. The evaluated procedure was, therefore, semi-automatic, and its results do not establish the performance of a fully autonomous processing pipeline. Future work will investigate automatic ROI selection from forest and building masks, together with association of the selected objects with mapped coastal features. The sea–land reference line could be extracted from the boundary between the corresponding segmentation masks, with additional geometric checks to reject occlusions and inconsistent boundaries. These steps were not implemented or evaluated in the present study.

The object height from the segmentation mask was compared with a manually determined reference pixel height. The manual measurement is denoted by $H_{p}^{\mathrm{man}}$, and the segmentation-based measurement by $H_{p}^{\mathrm{seg}}$.

The manually determined pixel height, $H_{p}^{man}$, is subject to measurement uncertainty and is used as a reference to assess the influence of segmentation on distance estimation. The resulting distances were compared with the GNSS-based distance, denoted by $D^{\mathrm{GNSS}}$. This task-oriented evaluation enabled direct assessment of how segmentation-boundary errors affected the final distance estimate.

For the building-based experiment, the segmentation mask was used to determine the highest visible point of the reference building. Because coastal objects partially occluded the lower part of the structure, its full vertical extent could not be measured directly. Therefore, the relevant image measurement was defined as the vertical distance between the manually determined sea–land reference line and the highest pixel assigned to the building.

Reference Objects

Accurate distance estimation depends on the visibility and detection of reference objects. In this study, the author assumes the AUV lacks high-resolution maps and instead uses simple maps or satellite imagery. Under these constraints, only objects with identifiable dimensions can serve as reference points.

Reference objects can be categorised (Table 4) by their physical dimensions, such as height and width, and by their frequency of occurrence. Examples include tall buildings, whose heights are available from public data, and natural features like forests, for which average heights can be estimated from regional studies. Other objects, such as cars and buses, as well as infrastructure, may also be used.

### 3.3. Geometric Distance-Estimation Model

Let $H_{r}$ denote the real-world vertical height of the reference object, $H_{p}$ its apparent vertical height in the image expressed in pixels, *f* the physical focal length of the camera, $N_{y}$ the vertical image resolution, and $h_{s}$ the physical height of the image sensor.

The physical pixel pitch in the vertical direction is

(1) $$p_{y}=\frac{h_{s}}{N_{y}},$$

where $p_{y}$ is expressed in millimetres per pixel. Under the pinhole-camera assumption, the distance to the reference object is estimated as

(2) $$D=\frac{H_{r}f}{H_{p}p_{y}}=\frac{H_{r}fN_{y}}{H_{p}h_{s}}.$$

An equivalent expression can be formulated using the vertical field of view $\theta_{v}$. The focal length expressed in pixels is

(3) $$f_{y}=\frac{N_{y}}{2\tan\left(\theta_{v}/2\right)}.$$

Consequently,

(4) $$D=\frac{H_{r}f_{y}}{H_{p}}=\frac{H_{r}N_{y}}{2H_{p}\tan\left(\theta_{v}/2\right)}.$$

Equations (2) and (4) assume that the reference-object plane is approximately perpendicular to the optical axis and that the measurement is performed using an undistorted image or sufficiently close to the optical axis.

### 3.4. Uncertainty of the Distance Estimate

The distance estimate is influenced not only by the segmentation error but also by the real-world object height and camera parameters. For the model given by Equation (2), the relative uncertainty may be approximated as

(5) $${\left(\frac{\sigma_{D}}{D}\right)}^{2}\approx {\left(\frac{\sigma_{H_{r}}}{H_{r}}\right)}^{2}+{\left(\frac{\sigma_{f}}{f}\right)}^{2}+{\left(\frac{\sigma_{N_{y}}}{N_{y}}\right)}^{2}+{\left(\frac{\sigma_{H_{p}}}{H_{p}}\right)}^{2}+{\left(\frac{\sigma_{h_{s}}}{h_{s}}\right)}^{2}.$$

Because the image resolution and sensor dimensions are fixed, the dominant components are generally the uncertainty of the real-world reference height $H_{r}$, the focal-length uncertainty, and the error in the apparent object height $H_{p}$. Equation (5) describes first-order uncertainty propagation under the assumption of uncorrelated input errors. The individual input uncertainties were not quantified sufficiently to construct a complete uncertainty budget or prediction intervals for individual distance estimates. The following sensitivity examples, therefore, illustrate the effects of parameter variations and should not be interpreted as statistical confidence bounds.

For small variations in the apparent object height, the resulting relative distance error can be approximated as

(6) $$\frac{\Delta D}{D}\approx -\frac{\Delta H_{p}}{H_{p}}.$$

At long distances, the reference object occupies only a small number of pixels, which causes quantisation of the estimated distance and increases the influence of individual segmentation-boundary errors. Atmospheric haze, reduced image contrast, refraction, camera motion, waves, and partial occlusion may further affect the apparent object height. The sensitivity expressed in Equation (6) also applies to pixel-height errors caused by lens distortion. If distortion changes the measured height from $H_{p}$ to $H_{p}\left(1+\varepsilon \right)$, while the other model parameters remain fixed, the estimated distance changes by a factor of 1/(1+ε). For example, a 1% increase in measured height produces approximately a 0.99% decrease in estimated distance. This example illustrates model sensitivity and does not represent a measured distortion level for the camera used in the experiments.

Camera pitch and roll may alter the apparent vertical extent of a reference object and the orientation of the reference line in the image, thereby affecting the distance estimate. Manual selection of the reference line does not itself compensate for these projection effects. Since camera attitude was not measured, the contribution of platform motion could not be isolated from the other error sources. Future AUV implementation would require evaluation of attitude compensation, image rectification, or camera stabilisation under representative wave conditions.

The influence of the Earth’s curvature depends on observation distance and camera height. For a camera approximately 1 m above the water, a spherical-Earth approximation neglecting atmospheric refraction gives a geometric horizon distance of approximately 3.57 km. Curvature-induced occlusion is, therefore, not expected over the distances investigated in Phase 2, which remained below 2 km. At the maximum distance of approximately 5.4 km in Phase 3, the minimum visible elevation above sea level is approximately 0.26 m. This is small relative to the height of the reference building, although its effect on the measured vertical extent also depends on the selected sea–land reference line and foreground occlusion. The Earth’s curvature may have a more substantial influence on the longer-range observations in Phase 1 and was not explicitly corrected for in the reported distance estimates.

The assumed forest height of 15 m is an approximate reference value rather than a measured height for each analysed coastal sector. Variations in species, age, and stand structure may, therefore, introduce a systematic error in the estimated distance. According to Equation (2), the estimated distance is directly proportional to the adopted reference height when the pixel measurement and camera parameters remain fixed. Changing the assumed height from 15 m to 14 m or 16 m would, thus, change the estimated distance by approximately −6.7% or +6.7%, respectively. This is a sensitivity example, not an empirically determined uncertainty interval.

Terrain elevation and changes in water level may additionally affect the correspondence between the assumed reference height and the vertical extent measured in the image. Waves may alter the visible lower boundary or temporarily obscure it. For a measurement referenced to sea level, a hypothetical 0.5 m change in an effective reference height of 15 m corresponds to an approximately 3.3% relative distance change under a first-order approximation. This is a sensitivity example, not a measured water-level variation. These effects should be distinguished from variations in physical tree height. Their individual contributions were not quantified in the present study, and forest-based results should, therefore, be interpreted as approximate distance estimates.

### 3.5. Primary Coastal Types Along the Baltic Sea

The Polish Baltic coastline exhibits diverse morphological features and vegetation types. Table 5 summarises representative coastal environments and the approximate forest reference heights considered in this study.

The approximate forest heights in Table 5 were derived from a preliminary analysis of digital surface model (DSM) and digital terrain model (DTM) data obtained from Geoportal [33,34]. Heights above ground were estimated as the difference between the DSM and DTM elevations. These values were used as approximate reference heights rather than site-specific field measurements.

Site-specific reference heights derived from elevation data could reduce reliance on approximate values assigned to broad coastal categories; this refinement was not evaluated in the present study.

## 4. Results and Discussion

The experimental evaluation assessed the semi-automatic distance-estimation procedure using coastal images acquired from a motorboat under maritime conditions. Deployment on an AUV and integration with a navigation system were not experimentally validated. The evaluation comprised three phases:

1. Phase 1: Single forest-region feasibility experiment.
  A single coastal-forest region was observed from 15 measurement positions located at different distances. The ROI was manually selected in each image to ensure that the same coastal sector was analysed.
2. Phase 2: Multiple forest-region experiment. Different coastal-forest regions were observed from 20 measurement positions distributed along an approximately 20 km-long coastal section. The analysed region was positioned close to the camera’s optical axis to reduce the influence of lens distortion.
3. Phase 3: Known-height building experiment. The Sea Towers building in Gdynia was used as a reference object of independently known height. Measurements were acquired from 27 positions located at distances ranging from 879 to 5383 m.

### 4.1. Phase 1

**Figure 3:**
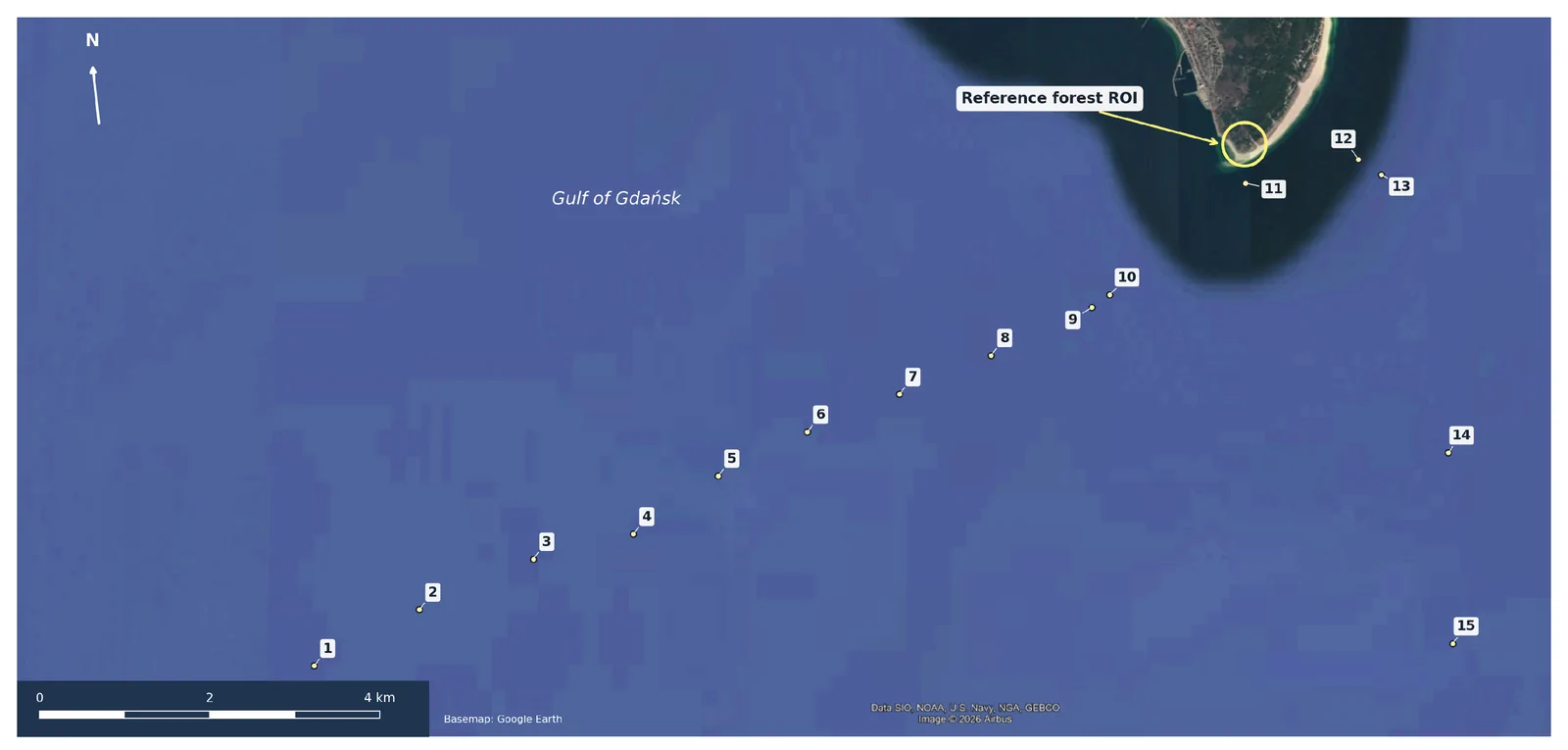
Measurement positions used in Phase 1 and the location of the selected coastal-forest region of interest.

**Figure 4:**
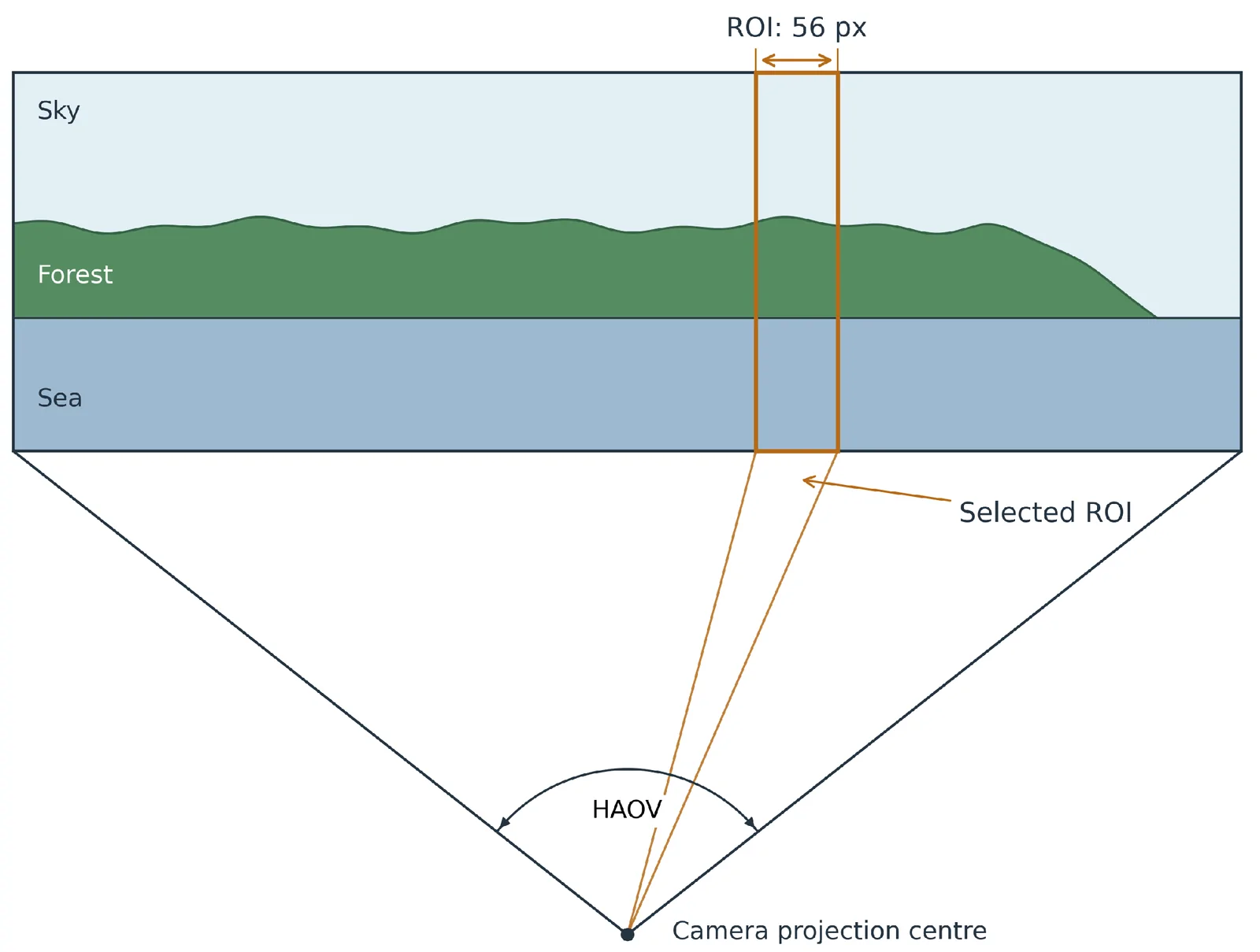
Definition of the 56-pixel-wide ROI used to determine the average apparent forest height. HAOV denotes the horizontal angle of view of the camera.

**Figure 5:**
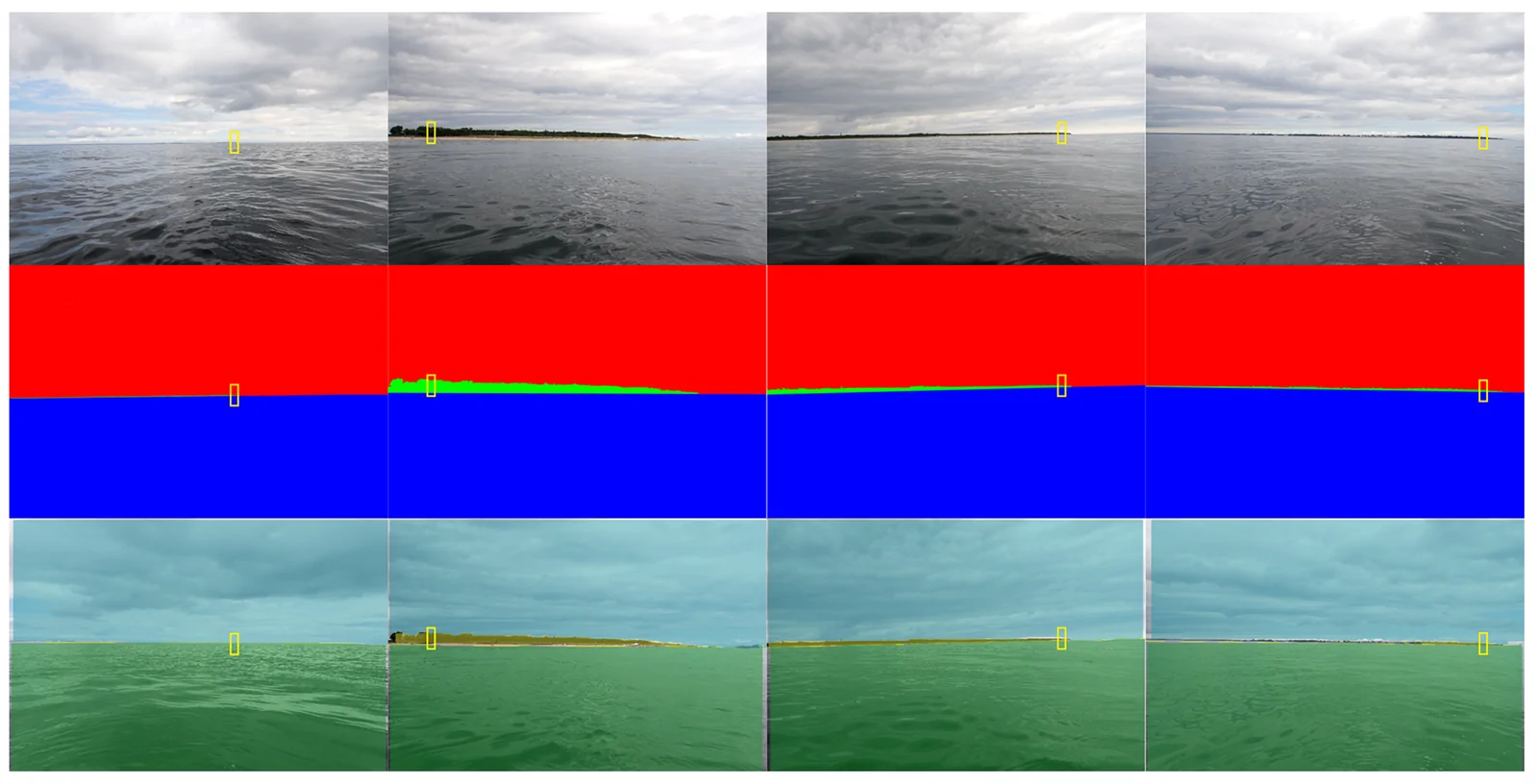
Original images (**top**), manually annotated reference masks (**middle**), and segmentation results (**bottom**). The yellow rectangles indicate the selected ROIs. The apparent forest height was determined by averaging the vertical extent of the forest mask across the analysed image columns within each ROI. Differences between the manual and predicted mask boundaries affect the pixel-height measurement used for distance estimation.

The first phase of the research investigated the feasibility of estimating distance to the shore using forest height in coastal areas. Measurements were taken at fifteen different points, covering distances of approximately 400 to 12,000 m to the ROI, as shown in Figure 3. The apparent forest height was determined by averaging the vertical pixel extent within a manually selected 56-pixel-wide ROI (Figure 4 and Figure 5). The ROI was selected to ensure that each distance estimate referred to the same coastal reference region. For the present study, it was assumed that the forest height would be 15 metres, as indicated in Table 5.

Table 6 compares the distances obtained using manually determined and segmentation-based pixel heights with the adopted reference distances. For several observations between approximately 1166 and 2606 m, estimates based on manual pixel measurements differed from the references by approximately 17–26 m. At the shortest reference distance of 369 m, the absolute difference was approximately 152 m. Potential contributors include the assumed forest height, local vegetation-height variation, optical distortion outside the central image region, and uncertainty in the adopted reference distance. Their individual contributions were not isolated.

At greater distances, the forest occupied only a small number of pixels. For example, at GNSS-based distances of 12,422 and 11,038 m, the manually measured forest height was five pixels in both images. The method, consequently, produced the same quantised distance estimate of 11,868 m. This illustrates that the available image resolution was insufficient to distinguish relatively small changes in distance at long range.

**Figure 6:**
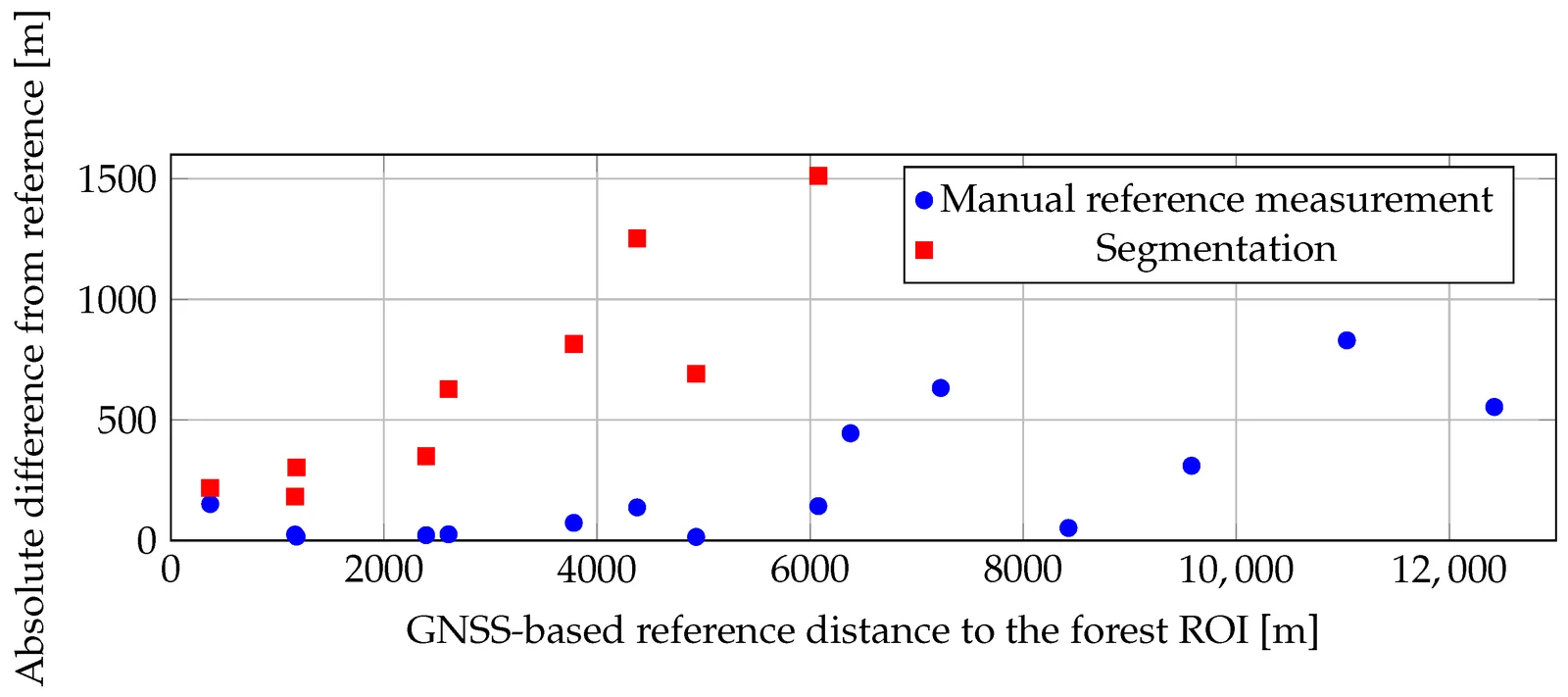
Absolute differences between the adopted reference distances and estimates obtained using manual pixel measurements and image segmentation, plotted against the reference distance to the selected forest ROI.

The segmentation-based estimates showed larger absolute differences from the adopted reference distances than estimates based on manual pixel measurements for the matched observations (Figure 6). For observations with an available segmentation-based estimate, the absolute differences ranged from approximately 183 to 1512 m. At distances exceeding approximately 6000 m, the forest could not be reliably segmented in several images.

Therefore, Phase 1 confirms the geometric feasibility of estimating distance from the apparent forest height, but it also demonstrates that image-segmentation accuracy and image quantisation substantially limit the operational range of the method. The long-range observations should, therefore, be interpreted as an exploration of the method’s limitations rather than evidence of reliable operation over the full tested range.

**Figure 7:**
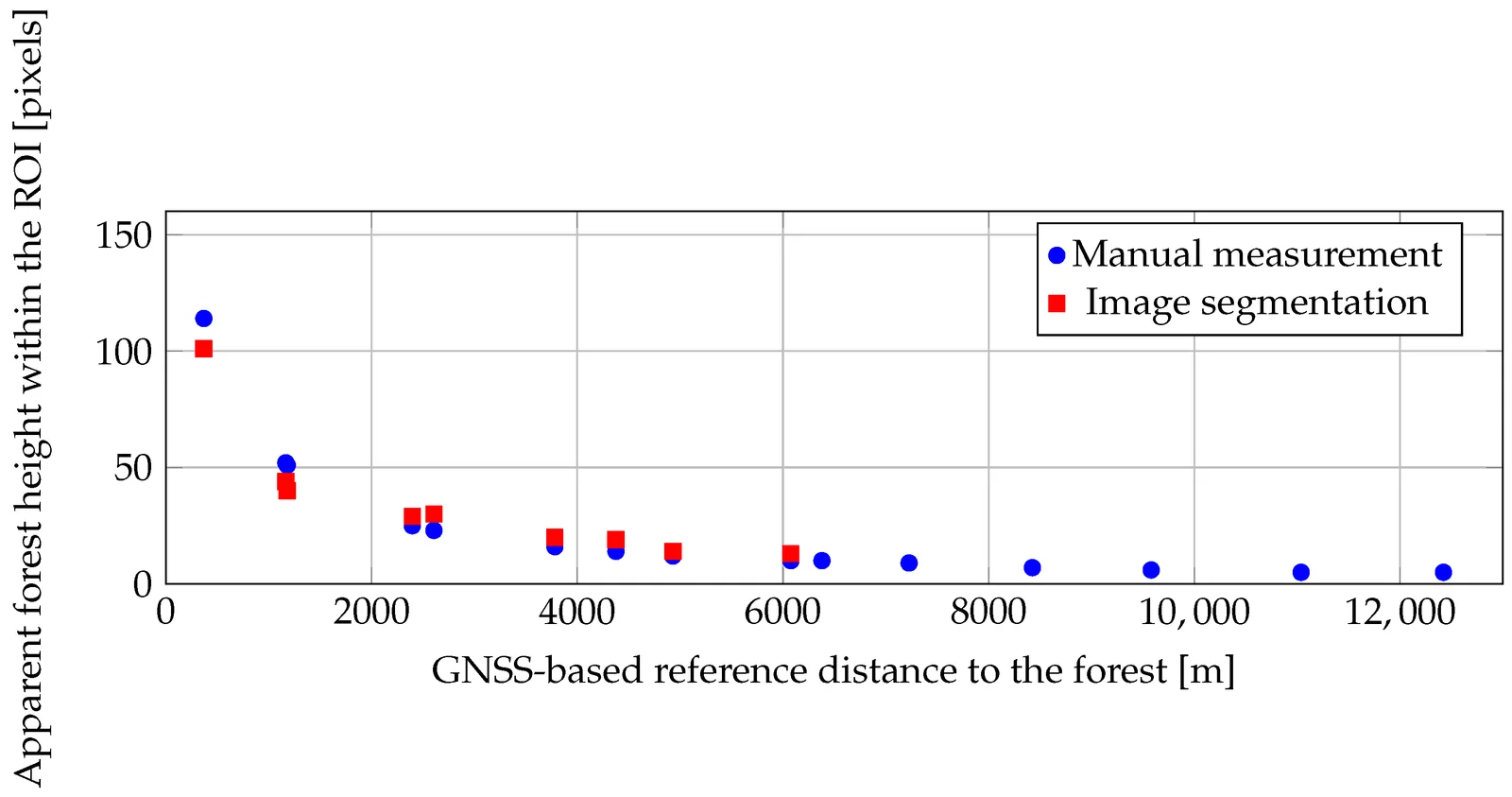
Relationship between the GNSS-based reference distance and the apparent forest height measured within the selected region of interest. Blue markers indicate manually determined reference measurements, whereas red markers indicate measurements obtained from image-segmentation masks.

Further analysis of the segmented images, compared with the manual reference measurement, revealed notable trends in terms of distance from the shoreline. At distances exceeding 2000 m, the applied segmentation model tended to over-segment, marking larger areas in the images than were present (Figure 7). At shorter distances, the segmentation model frequently underestimated the apparent forest height. Lens distortion may also have contributed to the distance-estimation errors, particularly for measurements away from the optical axis. However, its contribution could not be separated from reference-height uncertainty and other measurement errors without independent camera calibration.

### 4.2. Phase 2

**Figure 8:**
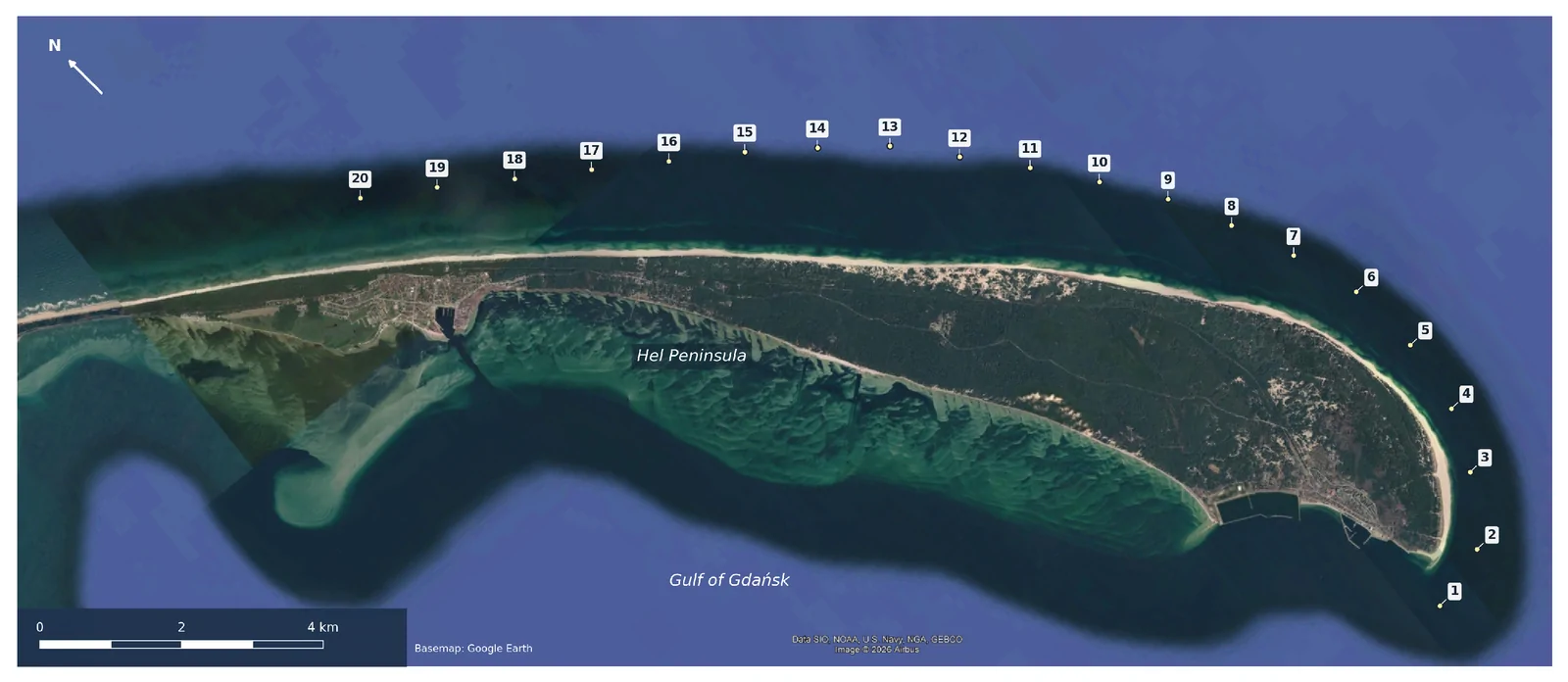
Measurement points used in Phase 2.

In the second phase of the experiments, a more detailed evaluation of the proposed method was conducted for distances ranging from approximately 500 m to 1900 m, as shown in Figure 8. Images were acquired at 20 measurement points distributed along an approximately 20 km-long section of the coast.

**Figure 9:**
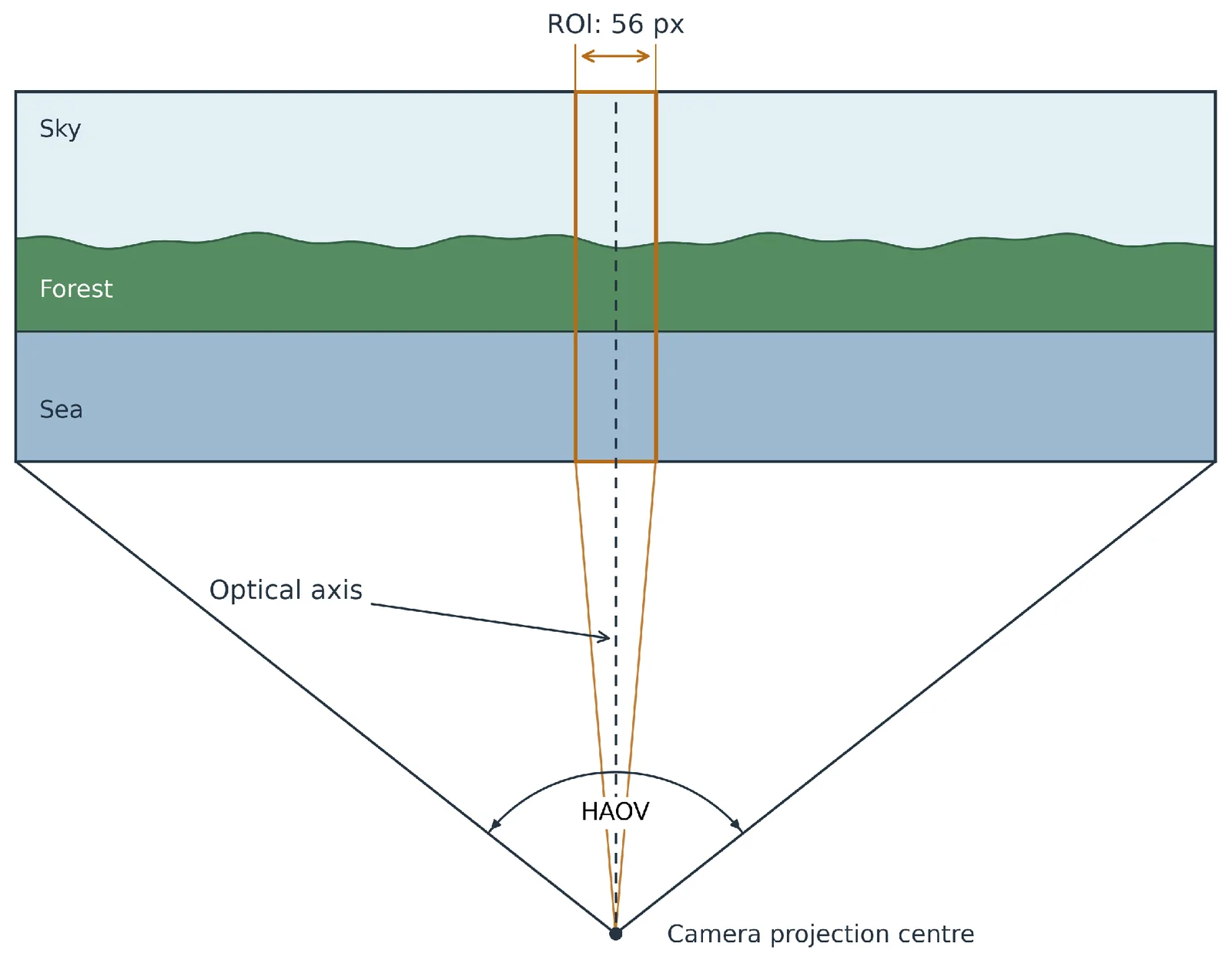
Selection of the 56-pixel-wide forest ROI near the optical axis in Phase 2. The apparent forest height was obtained by averaging the vertical extent of the forest mask across the analysed image columns within the ROI and was then used in Equation (2).

**Figure 10:**
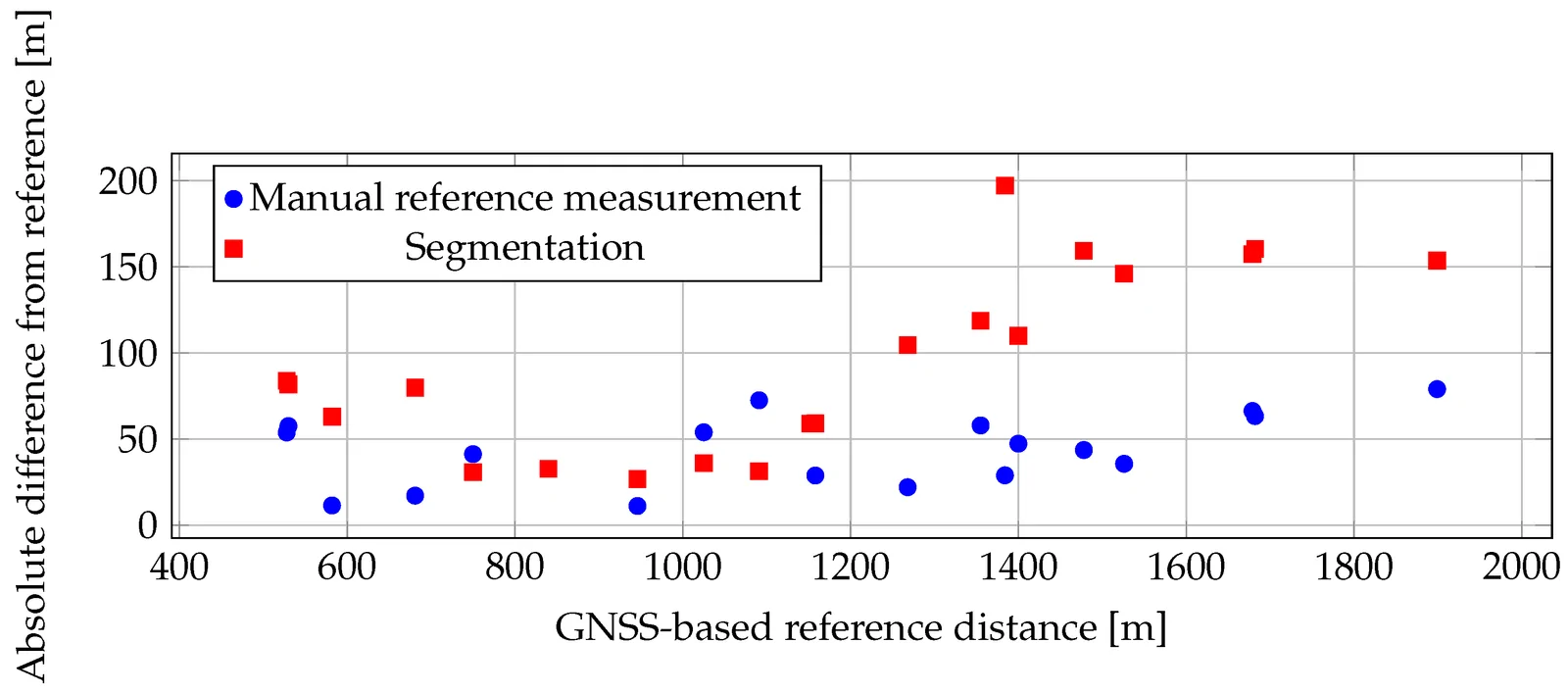
Absolute differences between estimated and adopted reference distances for Phase 2, corresponding to the results in Table 7.

To reduce the influence of optical distortions, the measurements were performed within a narrow region of interest located close to the camera’s optical axis, as illustrated in Figure 9. Estimates based on manual pixel measurements showed absolute differences from the adopted reference distances of approximately 11–79 m, while segmentation-based estimates showed differences of approximately 27–197 m (Figure 10, Table 7).

The uncertainty of the reference distances was not independently quantified. It may be significant relative to the smallest observed differences, particularly those of approximately 11–30 m. These values, therefore, indicate close agreement with the adopted references, but do not demonstrate absolute ranging accuracy at the same level. The contributions of reference uncertainty and estimation error cannot be separated using the available data.

The results support the feasibility of distance estimation over the investigated range of approximately 500–1900 m under the tested conditions. They do not establish a validated operational range or demonstrate compliance with specific AUV navigation-accuracy requirements. The relative sensitivity to pixel-height errors increases as the apparent forest height decreases.

### 4.3. Phase 3

**Figure 11:**
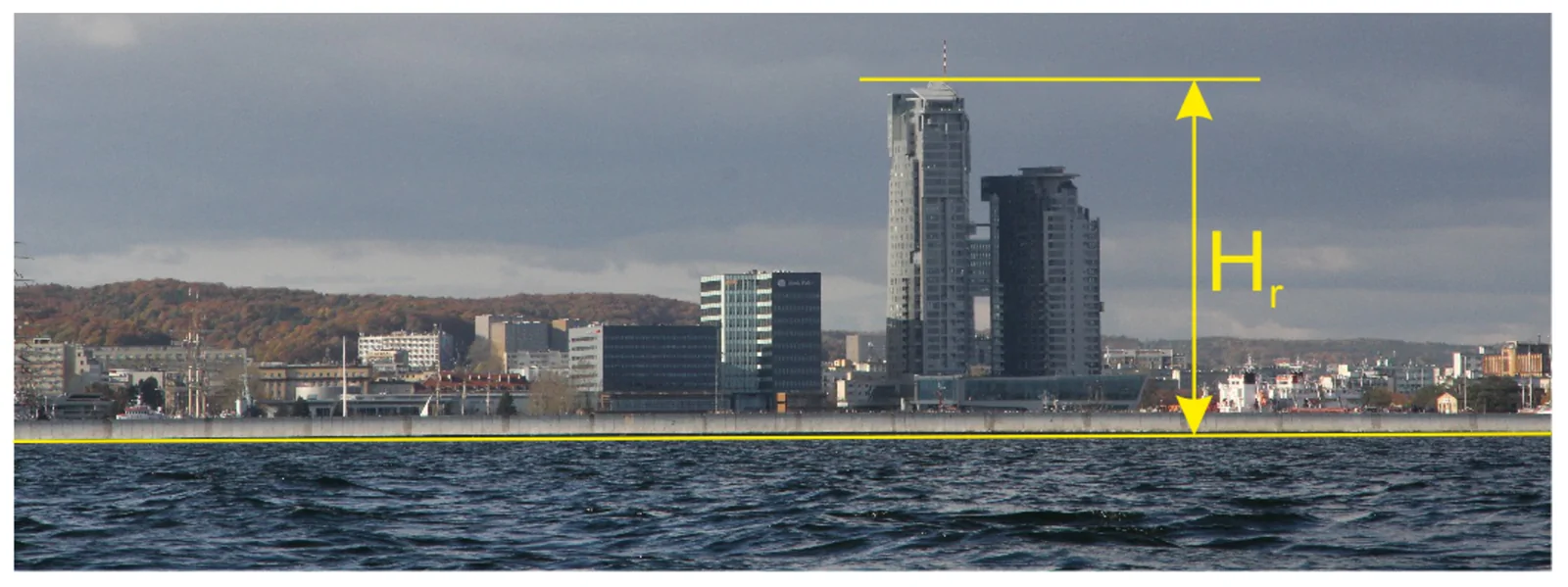
Reference building (Sea Towers in Gdynia).

**Figure 12:**
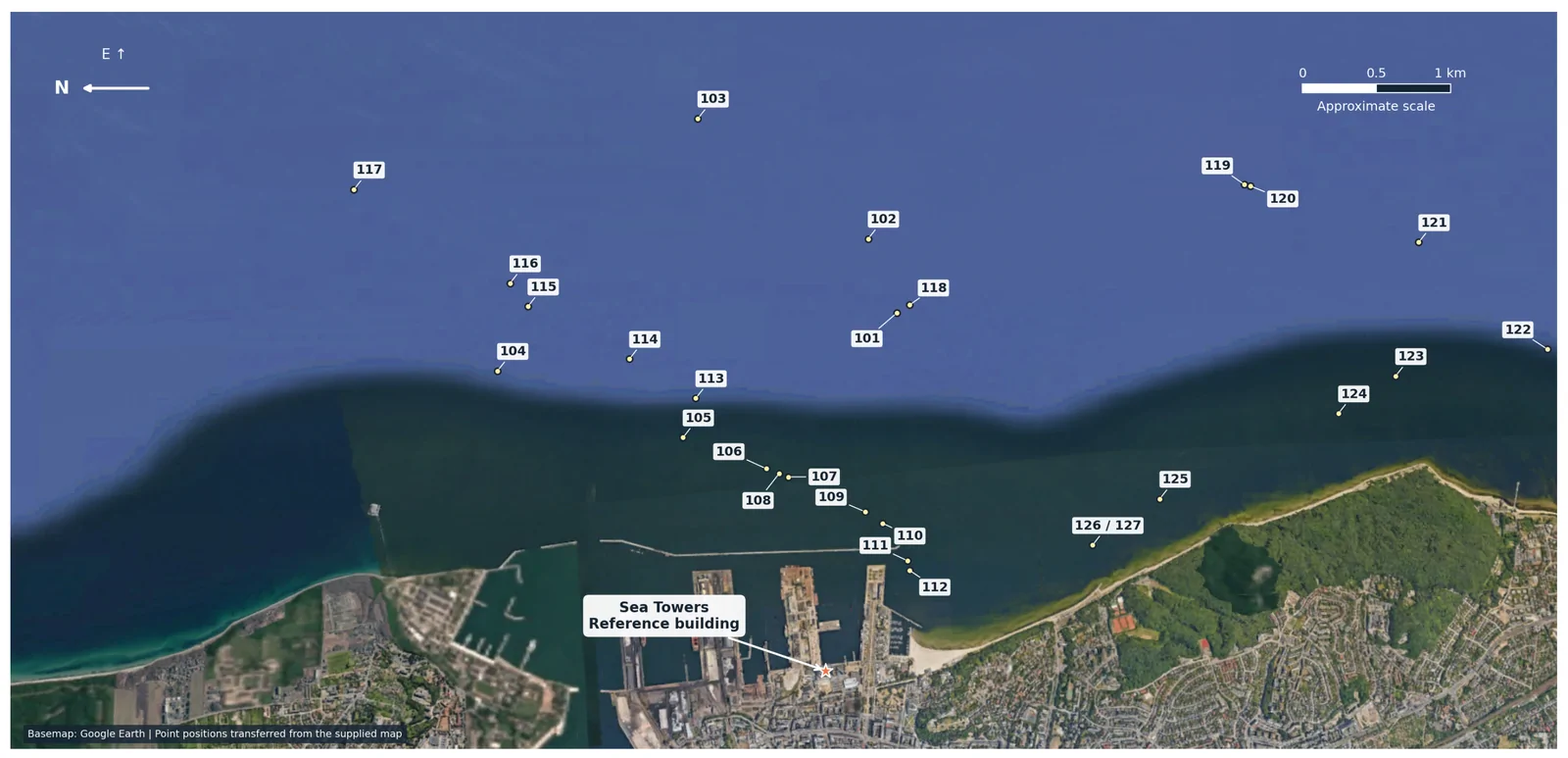
Measurement points used in Phase 3.

In the third phase of the experiment, the possibility of estimating the distance to a tall object of known height was investigated. The taller Sea Towers tower in Gdynia, with an adopted height of 125.4 m excluding the mast, was selected as the reference object, as shown in Figure 11. Measurements were acquired at 27 positions located between 879 and 5383 m from the building (Figure 12).

The building was partially occluded by coastal infrastructure and vegetation. Consequently, its complete silhouette and physical base were not visible from all measurement positions. The image measurement was, therefore, defined as the vertical distance between the sea–land reference line and the highest pixel assigned to the building class. The effective reference height was determined as

(7) $$H_{r}=H_{b}+H_{\mathrm{base}},$$

where $H_{b}$ denotes the physical height of the building and $H_{\mathrm{base}}$ denotes the elevation of its base above the adopted sea-level reference. The value $H_{\mathrm{base}}=2.5$ m was obtained from a digital elevation model. With $H_{b}=125.4$ m, the effective reference height was $H_{r}=127.9$ m for all observations.

The sea–land reference line was selected manually during this study. Therefore, the Phase 3 experiment did not constitute a completely automatic processing pipeline.

**Figure 13:**
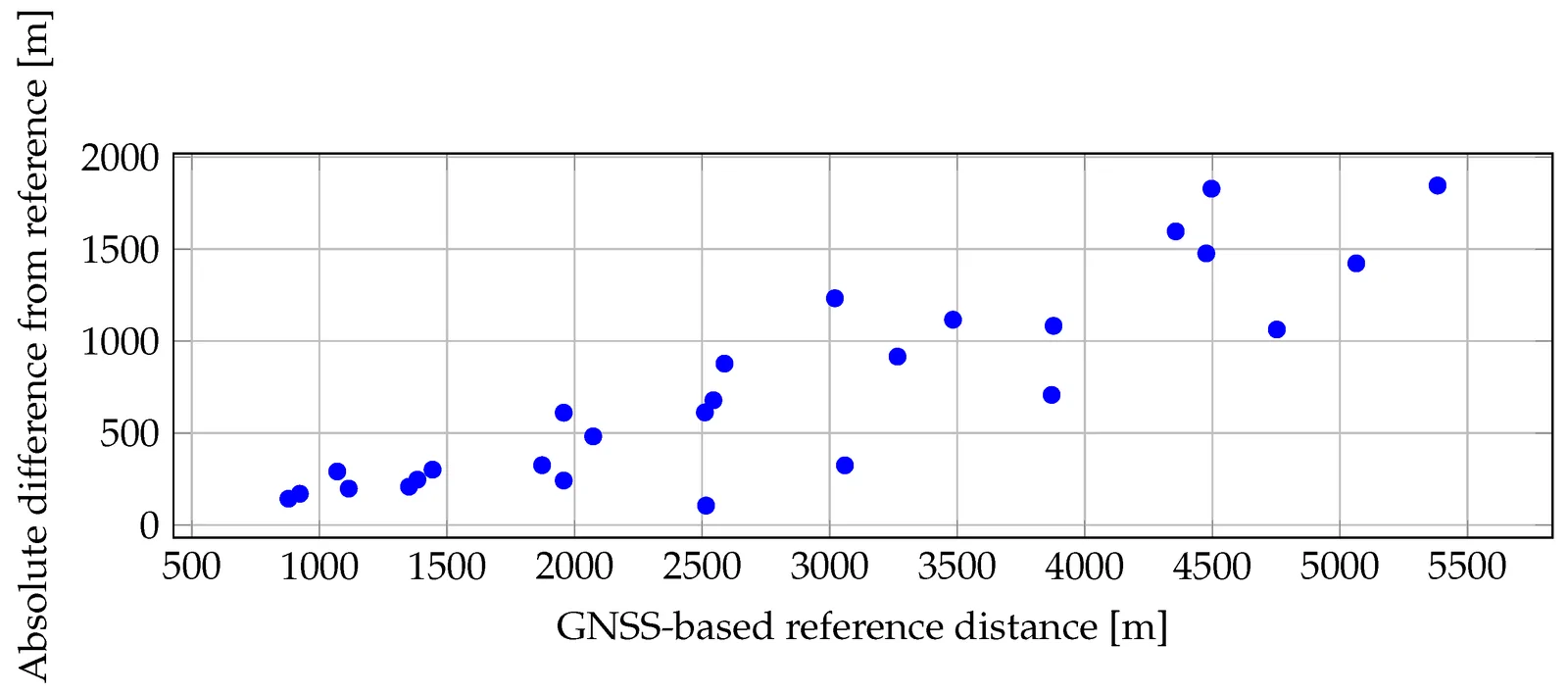
Absolute differences between estimated and adopted reference distances for the building-based experiment, corresponding to Table 8.

The results are presented in Table 8, Figure 8 and Figure 13. The mean absolute difference from the adopted reference distances was approximately 745 m. The maximum absolute difference was approximately 1846 m at a reference distance of approximately 5383 m (measurement point 122). The building occupied between 70 and 688 pixels in the vertical direction, depending on the measurement position.

Despite the building’s considerable apparent height, substantial differences from the adopted reference distances remained. Potential contributors include segmentation-boundary errors, uncertainty in the manually selected sea–land reference line, the adopted geometric assumptions, and uncertainty in the reference distances. The selected line may not coincide with the projection of the sea-level reference at the building’s location. The individual contributions of these effects were not quantified separately.

### 4.4. Summary of Experimental Results

Table 9 summarises the differences between the estimated distances and the adopted GNSS-based reference distances using mean absolute error (MAE), root mean square error (RMSE), median absolute error (MedAE), mean signed error (Bias), and mean absolute percentage error (MAPE). These conventional metric names are retained, but all metrics describe agreement with the adopted references, whose uncertainty was not independently quantified. They do not establish metrologically traceable absolute measurement accuracy.

Signed differences are defined as the estimated distance minus the adopted reference distance; negative bias, therefore, indicates underestimation relative to that reference. In Phase 1, segmentation-based estimates were available for 9 of the 15 observations. Manual results are reported both for all observations and for the same nine observations to enable a matched comparison. The Phase 3 results include manual selection of the sea–land reference line.

Operational summary.

- Phase 1: A single coastal-forest region. The evaluated reference-distance range was 369–12 422 m. Estimates based on manual pixel measurements were available for 15/15 observations, with a MAE of approximately 229 m and a MAPE of 5.72%. Segmentation-based estimates were available for 9/15 observations (60%), with a MAE of approximately 662 m and a MAPE of 25.38%. These nine observations covered 369–6077 m. For the same matched observations, manual measurements yielded a MAE of approximately 68 m and a MAPE of 6.03%. Principal limitations were the assumed forest height, pixel-height quantisation at long range, and the absence of usable segmentation-based estimates for six observations.
- Phase 2: Multiple coastal-forest regions. The evaluated reference-distance range was 528–1899 m. Estimates were available for 20/20 observations using both manual and segmentation-based pixel measurements. Manual measurements yielded a MAE of approximately 44 m and a MAPE of 4.21%; segmentation-based measurements yielded a MAE of approximately 95 m and a MAPE of 8.39%. Principal limitations were the assumed forest height and unquantified reference-distance uncertainty, which limits interpretation of the smallest observed differences.
- Phase 3: The Sea Towers building. The evaluated reference-distance range was approximately 879–5383 m. Segmentation-based estimates, with manual selection of the sea–land reference line, were available for 27/27 observations, with a MAE of approximately 745 m and a MAPE of 24.45%. Principal limitations were the manually selected reference line, its possible mismatch with the sea-level reference at the building’s location, and the adopted geometric assumptions.

Estimate availability describes only the analysed observations within the semi-automatic procedure. It is not an independently validated probability of successful autonomous operation. Reference-distance uncertainty and the absence of independent camera calibration affect interpretation in all three phases.

The Phase 2 results show lower segmentation-based errors than those obtained in Phases 1 and 3. However, the phases differ in distance range, reference objects, and measurement conditions, so these values should not be interpreted as a controlled comparison. The Phase 1 segmentation metrics describe only successful measurements and should be considered together with the six observations for which no usable estimate was obtained. The manual measurements provide a reference for assessing the influence of segmentation within the same ranging model. A comparative evaluation against alternative monocular-ranging methods on the same image dataset was not performed; the reported results, therefore, do not establish superiority over existing approaches.

## 5. Conclusions

This paper investigated the feasibility of a semi-automatic monocular image-based method for estimating distances to coastal reference objects, with a view to future application during temporary AUV surfacing. Apparent object heights obtained from image measurements were used in a geometric camera model.

The method was evaluated under real-world maritime conditions in three experimental phases comprising 15, 20, and 27 measurement positions. The reported differences and summary metrics describe agreement with adopted GNSS-based reference distances. Because the uncertainty of these references was not independently quantified and the camera geometry was not independently calibrated, the results do not establish metrologically traceable absolute measurement accuracy.

In Phase 2, segmentation-based absolute differences from the adopted references remained below 100 m for reference distances between approximately 500 and 1200 m and below 200 m over the full analysed range of approximately 500–1900 m. The smallest observed differences should be interpreted cautiously because reference-distance uncertainty may be significant relative to their magnitude.

At longer distances, small apparent object heights increased sensitivity to pixel quantisation and segmentation-boundary errors. In several Phase 1 images acquired beyond approximately 6000 m, the forest could not be reliably segmented. The building-based experiment covered reference distances of approximately 879–5383 m, with a MAE of approximately 745 m, a MAPE of 24.45%, and a maximum absolute difference of approximately 1846 m. These results indicate that a large apparent object height alone does not ensure close agreement with the adopted reference distances within the evaluated procedure.

The findings suggest that coastal reference objects could provide auxiliary distance information for future applications in GNSS-denied AUV navigation. The proposed method does not independently determine the complete vehicle position. Its output could be investigated as an additional observation for integration with inertial navigation, dead reckoning, map information, or observations of multiple coastal landmarks. Such integration was not experimentally validated in this study.

The main limitations include unquantified reference-distance uncertainty, uncertainty about the actual forest height, uncalibrated camera geometry, manual ROI and sea–land reference-line selection, segmentation errors, partial object occlusion, atmospheric visibility, unmeasured camera attitude and platform motion, image quantisation, and long-range geometric effects. Future work will focus on independent reference measurements, camera calibration, automatic landmark association, uncertainty-aware distance estimation, improved segmentation of small distant objects, and evaluation of integration with AUV navigation systems.

## Figures and Tables

**Table 1:** Principal parameters used for fine-tuning the segmentation network.

Parameter	Value
Network architecture	YOLOv8x-seg
Initialisation	Pre-trained weights
Input image size	640×640 pixels
Number of epochs	50
Batch size	8
Optimiser selection	Automatic (optimizer=auto)
Number of data-loading workers	8
Automatic mixed precision	Enabled
Deterministic training	Enabled
Random seed	0
Overlapping segmentation masks	Enabled
Mask downsampling ratio	4

**Table 2:** Data-augmentation parameters used during network training.

Augmentation Operation	Parameter Value
Hue modification	0.015
Saturation modification	0.7
Brightness modification	0.4
Translation	0.1
Scaling	0.5
Horizontal-flipping probability	0.5
Mosaic-augmentation probability	1.0
Random-erasing probability	0.4
Automatic-augmentation policy	RandAugment

**Table 3:** Class-wise mask average precision at an IoU threshold of 0.5 on the validation subset.

Class	Mask AP@0.5
Forest	0.570
Buildings	0.430
Sea	0.890
Sky	0.950

**Table 4:** Characteristics of various objects.

Object Name	Height (m)	Width (m)	Occurrence Frequency	Remarks
Car	approx. 1.5	1.5–6	Very rare	Only one region with visible vehicles
Bus	approx. 3.5	2.5–15	Very rare	
Single tree	1–30	1–15	Very rare	Trees predominantly appear in clusters or forests
Forest	10–30	–	Very high	
Buildings	5–200	5–130	Medium	
Window (door) in a building	0.5–5	0.5–5	Rare	
Building floor	approx. 3.2	–	Rare	
Tall structures (towers, lighthouses, chimneys)	Varies	Varies	Medium	Height information is widely available online

**Table 5:** Approximate forest reference heights for different coastal types along the Baltic Sea, based on a preliminary analysis of Geoportal DSM and DTM data.

Coastal Type	Dominant Vegetation	Approximate Reference Height (m)
Dune Coast (Sandy)	Pine, birch	12
Cliff Coast	beech, oak, mixed forest	20
Barrier Spits and Lagoon Shores	Pine, alder	15
Lagoon and Deltaic Coast	Alder, willow, poplar, mixed forest	17
Low-Lying Forested Coast	Pine, oak, birch	16

**Table 6:** Comparison of the GNSS-based reference distance with distances estimated using manually determined and segmentation-based forest heights.

Measurement Point	GNSS-Based Distance to the Forest ROI [m]	Manual Forest Height [Pixels]	Segmentation-Based Forest Height [Pixels]	Distance Estimated from Manual Measurement [m]	Distance Estimated from Segmentation [m]	Absolute Difference from Reference [m] (Manual Measurement)	Absolute Difference from Reference [m] (Segmentation)
1	12,422	5	N/A	11,868	N/A	554	N/A
2	11,038	5	N/A	11,868	N/A	830	N/A
3	9580	6	N/A	9890	N/A	310	N/A
4	8425	7	N/A	8477	N/A	52	N/A
5	7226	9	N/A	6593	N/A	633	N/A
6	6077	10	13	5934	4565	143	1512
7	4930	12	14	4945	4239	15	691
8	3782	16	20	3709	2967	73	815
9	2606	23	30	2580	1978	26	628
10	2396	25	29	2374	2046	22	350
11	369	114	101	521	588	152	219
12	1166	52	44	1141	1349	25	183
13	1180	51	40	1164	1484	17	304
14	4376	14	19	4239	3123	137	1253
15	6379	10	N/A	5934	N/A	445	N/A

**Table 7:** Phase 2 estimated distance compared to GNSS measurements.

Distance to Forest [m] (GNSS)	Number of Pixels (Manual Reference Measurement)	Number of Pixels (Segmentation)	Estimated Distance [m] (Manual Reference Measurement)	Estimated Distance [m] (Segmentation)	Absolute Difference from Reference [m] (Manual Measurement)	Absolute Difference from Reference [m] (Segmentation)
528	102	97	582	612	54	84
530	101	97	588	612	58	82
582	100	92	593	645	11	63
681	85	78	698	761	17	80
750	75	76	791	781	41	31
840	68	68	873	873	33	33
946	62	61	957	973	11	27
1025	55	60	1079	989	54	36
1091	51	56	1164	1060	73	31
1152	49	49	1211	1211	59	59
1158	50	54	1187	1099	29	59
1268	46	51	1290	1164	22	105
1355	42	48	1413	1236	58	119
1384	42	50	1413	1187	29	197
1400	41	46	1447	1290	47	110
1478	39	45	1522	1319	44	159
1526	38	43	1562	1380	36	146
1679	34	39	1745	1522	66	157
1682	34	39	1745	1522	63	160
1899	30	34	1978	1745	79	154

**Table 8:** Estimated distance compared to GNSS measurements.

Measurement Point	Apparent Building Height [Pixels]	GNSS-Based Reference Distance [m]	Estimated Distance [m]	Absolute Difference from Reference [m]
101	193	2516	2622	106
102	283	3021	1788	1233
103	160	3870	3162	708
104	185	3060	2735	325
105	327	1873	1547	326
106	443	1444	1142	301
107	445	1384	1137	247
108	443	1351	1142	209
109	552	1115	917	198
110	650	1070	778	292
111	672	923	753	170
112	688	879	735	143
113	198	2073	2555	482
114	157	2544	3223	678
115	121	3266	4182	916
116	110	3484	4600	1116
117	87	4753	5816	1063
118	146	2588	3466	878
119	85	4476	5953	1476
120	80	4497	6325	1828
121	78	5064	6487	1423
122	70	5383	7228	1846
123	85	4357	5953	1596
124	102	3878	4961	1083
125	162	2511	3123	612
126	230	1958	2200	242
127	197	1958	2568	611

**Table 9:** Summary statistics of differences between estimated and adopted reference distances. Only observations with an available estimate are included. Bias is the mean estimated distance minus the adopted reference distance. The uncertainty of the reference distances was not independently quantified.

Phase	Measurement Method	*n*	MAE	RMSE	MedAE	Bias	MAPE
			**(m)**	**(m)**	**(m)**	**(m)**	**(%)**
1	Manual, all observations	15	229	343	137	−48	5.72
1	Manual, matched observations	9	68	88	26	−31	6.03
1	Segmentation	9	662	795	628	−505	25.38
2	Manual	20	44	48	46	44	4.21
2	Segmentation	20	95	108	83	−49	8.39
3	Segmentation	27	745	918	612	437	24.45

## Data Availability

The data that support the findings of this study are available from the corresponding author upon reasonable request.

## Abbreviations

The following abbreviations are used in this manuscript:

3D	Three-dimensional
AI	Artificial Intelligence
AOV	Angle of View
ASV	Autonomous Surface Vehicle
AUV	Autonomous Underwater Vehicle
CNN	Convolutional Neural Network
DEM	Digital Elevation Model
DVL	Doppler Velocity Log
GNSS	Global Navigation Satellite System
HAOV	Horizontal Angle of View
IMU	Inertial Measurement Unit
MAV	Micro Aerial Vehicle
MLO	Mine-Like Objects
MSA	Maritime Situational Awareness
ROI	Region of Interest
SLAM	Simultaneous Localisation and Mapping
TAN	Terrain-Aided Navigation
TBN	Terrain-Based Navigation
UAV	Unmanned Aerial Vehicle
USV	Unmanned Surface Vehicle
VO	Visual Odometry
vSLAM	Visual Simultaneous Localisation and Mapping
